# Doxorubicin-induced transcriptome meets interactome: identification of new drug targets

**DOI:** 10.3906/biy-2107-45

**Published:** 2021-12-20

**Authors:** Hilal TAYMAZ-NİKEREL

**Affiliations:** Department of Genetics and Bioengineering, Faculty of Engineering and Natural Sciences, İstanbul Bilgi University, İstanbul, Turkey

**Keywords:** Bioinformatics, doxorubicin, interactome, systems biology, transcriptome

## Abstract

The working mechanism of the chemotherapeutic drug doxorubicin, which is frequently used in cancer treatment, its effects on cell metabolism, and pathways activated solely by doxorubicin are not fully known. Understanding these principles is important both in improving existing therapies and in finding new drug targets. Here, I describe a systems-biology approach to find a generalizable working principle for doxorubicin by superimposition of human interactome over gene datasets commonly expressed among various cancer types. The common –in at least two different diseases–transcriptional response of distinctive cancer cell lines to doxorubicin was reflected via 199 significantly and differentially expressed genes, mostly related to the regulation of transcription. Then, by integrating with interactome data, an active network was constructed allowing detection of clusters. Since each cluster defines densely connected regions, another level of understanding of functional principles is provided. Significant clusters were associated with the linked transcription factors and transcriptional factor enrichment analysis within these regulatory networks led to the proposition of Pou5f1b, Znf428, Prmt3, Znf12, Erg, Tfdp1, Foxm1, and Cenpa as new drug targets in drug development that can be applied in different cancer types.

## 1. Introduction

One of the most used chemotherapeutic drugs, shown to be efficient in different types of cancer, is doxorubicin. Doxorubicin is known to intercalate into DNA, inhibit topoisomerase II, lead to DNA double-strand breaks, induce production of reactive oxygen species (ROS), overproduce ceramide, damage chromatin through histone modifications and disrupt nucleosome assembly (Yang et al., 2014; van der Zanden et al. 2020). Although it has a high drug efficacy, usage of doxorubicin creates resistance and causes several side effects (Ferreira et al., 2017; Ashrafizaveh et al., 2021). Despite the known functions of doxorubicin, the mechanism of action is not fully clarified. To overcome the limitations of the drug, to design better therapies and to utilize it in combination therapies, comprehensive understanding of the mechanism is required.

With the broad usage of transcriptome profiling, there have been studies suggesting signature genes that can be used to predict type of a disease, subtype and eventually the drug that can be administered for therapy, centering at cancer- and/or cell type-specific responses (Yu et al., 2019; Gopi and Kidder, 2021) or effects of various concentrations of the drug on a certain cell model (Maillet et al., 2016). Functional annotation and/or correlation analysis together with clustering methods on gene expression data are used to identify differences between tumors or cell lines. Focusing on the differences in gene expression, therefore, serves to improve the prediction of that certain type of disease’s response. However, such approaches yield ad hoc results specific to the used data and/or platform used for the measurement of data leading to lack of generalization.

Integrative multiomic approaches were applied to understand doxorubicin-induced cardiotoxicity, to identify the common signature of anthracycline-induced cardiotoxicity, or to suggest patient-specific optimal combination therapy (Selevsek et al., 2020; Cava et al., 2021). Systems-wide effects of doxorubicin have been investigated also in yeast cells. While long-term treatment showed extensive reconfiguration of metabolic and signaling networks with ROS formation and DNA damage (Taymaz-Nikerel et al., 2018), short-term treatment showed the contribution of DNA repair, DNA replication, and RNA surveillance pathway (Karabekmez et al., 2021). Addition to the transcriptome and fluxome, interactome was integrated in those studies, which explained well the effects in eukaryotic yeast cells. Mentioned metaanalysis studies focused on the effects of doxorubicin in a certain cell type or differences across the set of various cells. However, a genome-wide approach to explore the common response of doxorubicin in different cancer types has not been applied.

Among the cancer types in which doxorubicin treatment has been applied either in single form or in combination with other chemotherapeutic drugs, are breast cancer (Di Francesco et al., 2021), leukemia (Turk et al., 2020), and nonsmall cell lung cancer (Shahriari et al., 2021). Though, doxorubicin is not much preferred in the treatment of colon cancer (Sonowal et al., 2017) or renal cancer (Acharya and Singh, 2021) due to severe cardiotoxiciy and drug resistance. These different sets of diseases are selected to examine the common effect(s) of doxorubicin regardless of the cancer/cell type. By this, affected processes, drug-specific transcriptional and regulatory networks in doxorubicin treatment irrespectively can be identified, and consensus mechanism of action can be offered.

Here, a systems biology approach is described, which is based on the integration of transcriptome with interactome data for the selected cell types, providing the regulatory components having a role in the mechanism of action of doxorubicin. Enrichment analyses relative to processes, pathways, and transcription factors demonstrate that there are several common types of machinery responsible for the cellular changes caused by doxorubicin. Moreover, such an approach enables the prediction of new targets for the development of drugs or of combination therapies.

## 2. Materials and methods

### 2.1. Compilation of gene expression data

While selecting gene expression datasets, cancer types, cell lines, dosage, and duration of administration of doxorubicin drug were taken into consideration. Microarray data, obtained using the Affymetrix platform, associated with doxorubicin was collected by scanning the NCBI Gene Expression Omnibus, GEO, and ArrayExpress, the functional genomics data repository supporting MIAME-compliant data submission.

Doxorubicin-induced transcriptome responses were then collected from the measurements across the National Cancer Institute (NCI)-60 cell line panel (Monks et al., 2018). In that study, expression of genes in NCI-60 human tumor cell lines were measured in response to several anticancer agents for 2, 6, and 24 h. For renal cancer, breast cancer, leukemia, nonsmall cell lung cancer and colon cancer, the gene expression data, Gene Expression Omnibus (GEO) accession number of GSE116441 measured in the cells treated with 1000 nm of doxorubicin were used. As reference gene expressions, data measured in untreated control cultures were applied. Details of the compiled data are given in Supplementary Table S1.

### 2.2. Data analysis

#### 2.2.1. Differential expression analysis in transcriptome

First, datasets were normalized. Differentially expressed genes (DEGs) were defined from normalized log-expression values using linear models for microarray data. In data analysis, t-test method was applied to determine statistically significant (fold change in gene expression >|1.05|, p-value < 0.05) subsets. These analyses were performed in CLC Genomics Workbench (Qiagen Bioinformatics).

#### 2.2.2. Integration with interactome data

A protein-protein interaction (PPI) network was created using the proteins encoded by the common — in at least two diseases — differentially expressed genes and physical interactions of these proteins obtained from BIOGRID Homo sapiens 3.5.187 database comprising 613869 protein-protein interactions (Oughtred et al., 2019). Subnetwork analysis was carried out to detect clusters within the active network via MCODE application in Cytoscape 3.8.0, where all networks were visualized (Shannon et al., 2003).

#### 2.2.3. Functional annotation analysis

Functional annotation analyses were carried out for the common differentially expressed genes and for each cluster identified within the active PPI network. Gene ontology (GO) term enrichment analyses were performed in DAVID functional annotation tool to identify significantly (Benjamini-Hochberg corrected p-value < 0.05) associated biological process, molecular function, cellular compartment, and KEGG pathways (Huang et al., 2009).

#### 2.2.4. Transcription factor enrichment analysis

For each cluster identified within the active PPI network, transcription factor (TF) enrichment analysis was done in ChEA3, a web-based tool, ranking TFs (Keenan et al., 2019).

## 3. Results and discussion

This study aims to explain the working principle of doxorubicin and, via this, propose new drug targets for cancer therapy. A systems biology approach was applied through a metaanalysis of available transcriptome data measured in the presence of doxorubicin subjected to different cell lines of cancer. Commonly altered genes were coupled with interactome yielding a mechanistic and systems-level understanding.

### 3.1. Common genes differentially expressed under doxorubicin treatment in different cancer types

**Figure 1:**
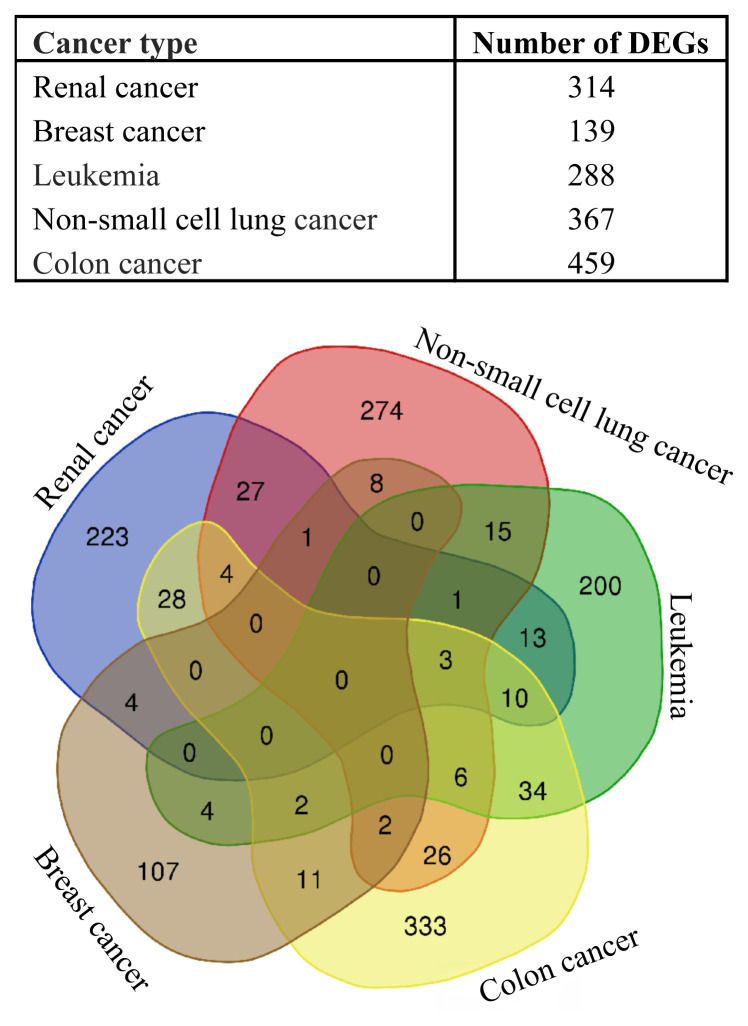
Number of identified differentially expressed genes (DEGs) in the presence of doxorubicin exposed to different cancer cell lines.

Genome-wide response of renal cancer, breast cancer, leukemia, nonsmall cell lung cancer, and colon cancer cells to doxorubicin at the transcriptional level revealed that numerous genes were differentially and significantly (fold change >|1.05| and p-value <0.05) expressed. Number of identified differentially expressed genes (DEGs) for each cancer type are presented in Figure 1.

**Figure 2:**
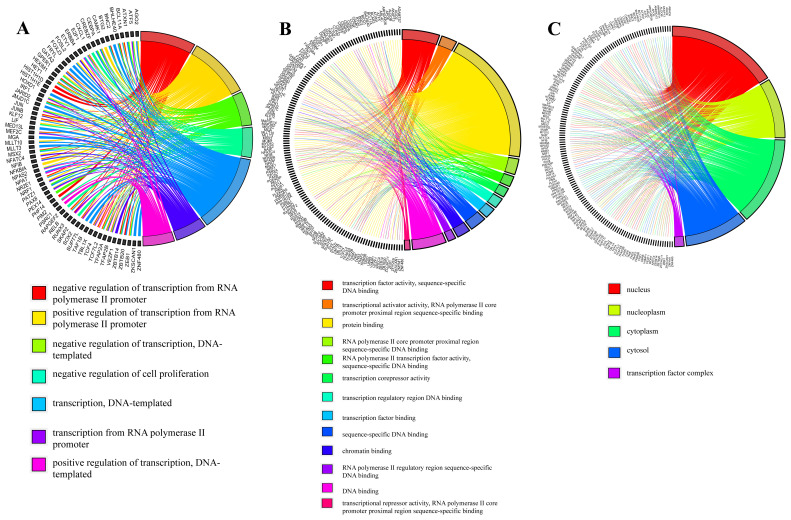
Functional enrichment analysis among the common 199 differentially expressed genes. Plots are generated in R via GOplot: the left side of the circles display genes, and the right sides display GO terms, annotated below each circle. If the gene belongs to a GO term, there is a line between the gene and the GO term for biological processes (A), molecular functions (B), and cellular compartments (C).

In order to determine the common DEGs of different cancer types, genes that showed significant differences in at least two diseases were identified. This resulted in 199 genes, listed in Supplementary Table S2. Gene ontology (GO)-term enrichment analyzes for these 199 common DEGs revealed statistically significant (Benjamini-Hochberg < 0.05) biological processes (Figure 2A), molecular functions (Figure 2B), and the cellular part they take place in (Figure 2C). TNF signaling pathway and osteoclast differentiation are significant pathways. When these findings are examined, it is seen that these common genes are mostly related to the regulation of transcription (Figure 2A).

### 3.2. Construction of the active network

An active network was formed by obtaining the protein-protein interactions of the proteins encoded by the common 199 DEGs (Supplementary Table S2). After removing the unlinked residues, a linked network of active protein-protein interactions was identified through 7022 proteins and 15775 protein-protein interactions. Topological analysis of the active protein-protein interaction network revealed that it is a scale-free network, with degree distribution following the power law model P(k) ≈ k^−0.856^ with R^2^ = 0.975, (Supplementary Table S3 and Figure S1).

#### 3.2.1. Modular analysis of the active network

**Figure 3:**
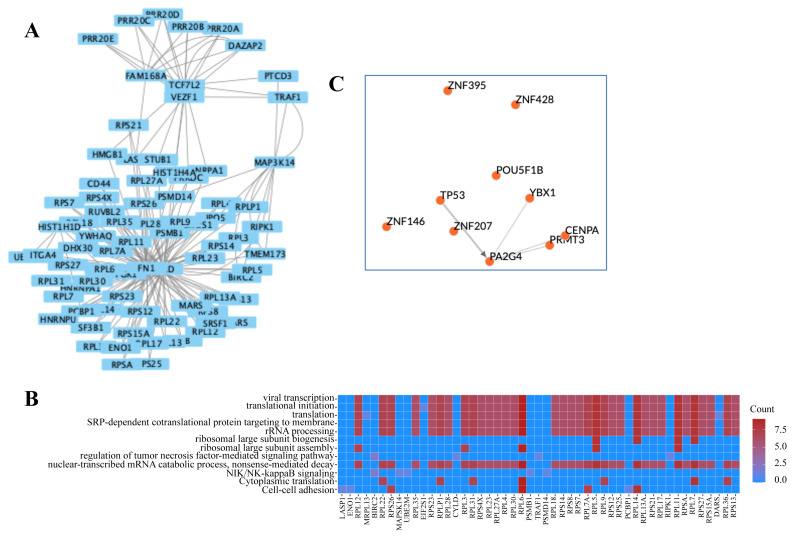
Modular representation of Cluster 1 (A), significant (Benjamini-corrected p-value < 0.05) biological process gene ontology (GO) terms in Cluster 1 (B), transcription factor enrichment for Cluster 1 (C).

The heavily connected clusters in the active network were determined in MCODE application, resulting in nine clusters (Supplementary Figure S2). The parameters for each cluster are summarized in Supplementary Table S4. Functional enrichment analyzes were performed for the identified clusters. Biological process terms/cellular compartments/molecular functions and pathways that were found to be statistically significant (Benjamini-Hochberg p-value < 0.05) are presented for the first three clusters (Figures 3A).

Among the biological processes related to the genes/proteins in Cluster 1, besides general terms such as transcription and translation, more specific processes such as ribosomal large subunit, ribosomal large subunit biogenesis, NIK/NF-kappaB signaling, tumor necrosis factor-mediated regulation of signaling pathway, and cell adhesion were also observed (Figure 3B). NIK/NF-kappaB was reported to potentially trigger doxorubicin resistance (Gao et al., 2019). Ribosomes were frequently encountered in different terms in this cluster. This allows us to predict in advance that Cluster 1 is important in defining transcriptional regulatory networks.

**Figure 4:**
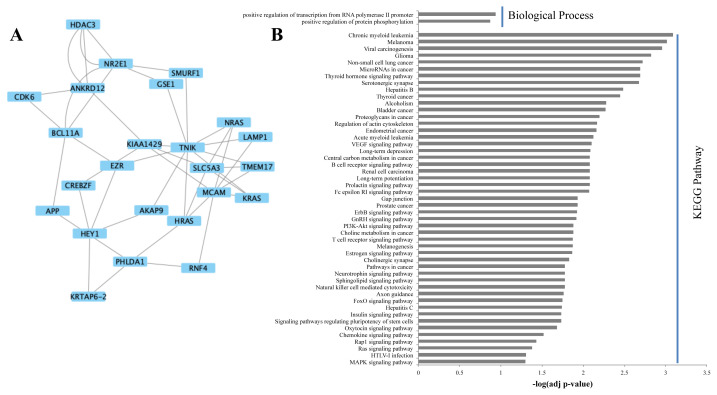
Modular representation of Cluster 2 (A), significant (Benjamini-corrected p-value < 0.05) biological process gene ontology (GO) terms and KEGG pathways in Cluster 2 (B).

In Cluster 2 (Figure 4A) there are no significant (Benjamini-Hochberg p-value < 0.05) results for biological processes, but many for pathways (Figure 4B). Many cancer-related terms have been obtained, such as nonsmall cell lung cancer, glioma, viral carcinogenesis, melanoma, chronic myeloid leukemia, microRNAs in cancer, serotonergic synapse, thyroid hormone signaling pathway, thyroid cancer, bladder cancer, proteoglycans in cancer, regulation of actin cytoskeleton, endometrial cancer, acute myeloid leukemia, central carbon metabolism in cancer, renal cell carcinoma, prostate cancer, and choline metabolism in cancer. In addition, the genes in Cluster 2 were found to be associated with several signaling pathways that can be associated with cancer, long-term depression, and natural killer cell-mediated cytotoxicity.

**Figure 5:**
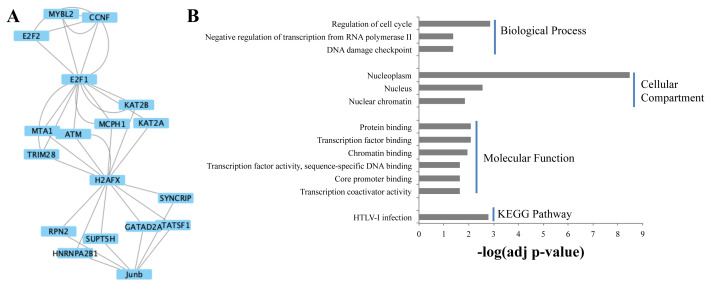
Modular representation of Cluster 3 (A), significant (Benjamini-corrected p-value < 0.05) biological process, molecular function, cellular compartment gene ontology (GO) terms, and KEGG pathways in Cluster 3 (B).

Statistically significant biological processes associated with the genes/proteins that constitute Cluster 3 (Figure 5A) are cell cycle regulation, DNA damage checkpoint, and negative regulation of transcription from the RNA polymerase II promoter (Figure 5B). Significant molecular function terms are mostly related to binding. HTLV-I infection is the only significant pathway in Cluster 3.

#### 3.4.2. Transcriptional regulatory networks

Identification of the transcription factors (TFs) responsible for the regulation of genes, which had altered expression is important to understand the working mechanism of doxorubicin. Transcription factor enrichment analysis for the three statistically significant clusters was performed in ChEA3 (Keenan et al., 2019). TF co-expression networks help visualize top-ranked transcription factors in the framework of the larger human transcription regulation network. TF-TF co-regulatory networks are dynamically created using the best results of the selected library. The regulatory network for the top 10 TF results for Cluster 1 is presented in Figure 3C.

Expression of TF Pou5f1bp associated with Cluster 1 was shown to increase in cervical cytology and was proposed as a biomarker to identify cervical high-grade squamous lesions (Chen et al., 2020). Another TF, Znf428p, was presented as one of the regulators of cell invasion and cell migration in tumor cells (Barreiro-Alonso et al., 2018); it is also one of the seven panel biomarker candidates for the early diagnosis of hepatocellular carcinoma (Zhang et al., 2020). The transcription factor Prmt3p (protein arginine methyltransferase 3) has been shown to be dysregulated in gemcitabine-resistant pancreatic cancer cells. Overexpression of Prmt3p resulted in increased resistance to gemcitabine in pancreatic cancer cells, while reduction of Prmt3p was observed to restore gemcitabine sensitivity in resistant cells (Hsu et al., 2018). Based on this, inhibition of Prmt3p was proposed as a new strategy for the treatment of gemcitabine-resistant pancreatic cancer (Hsu et al., 2018).

The regulatory network for the top 10 TFs of Cluster 2 is presented in Supplementary Figure S3. Znf12p was proposed as a new target in imatinib-resistant gastrointestinal stromal tumor cells (Cao et al., 2018). Znf888p was reported as one of the methylation-based genes associated with clear-cell renal cell carcinoma (Wang et al., 2020). Overexpression of another Cluster 2-related TF, Ergp, was reported to be associated with tumor stage in prostate cancer but did not strongly predict the rate of relapse or death among men treated with radical prostatectomy (Pettersson et al., 2012). TMPRSS2-ERG gene fusions are the major subtype of prostate cancer and are predominantly seen in young patients and lead to constitutive overexpression of the transcription factor Ergp. ERG overexpression alone is not prognostic; however, ERG was shown to modulate the expression of > 1600 genes in prostate epithelial cells (Büscheck et al., 2019).

The regulatory network for the top 10 TFs of Cluster 3 is presented in Supplementary Figure S4. Of the TFs associated with Cluster 3, Tfdp1p has the highest mean-rank. Extensive characterization of DNA amplification at chromosome region 13q34 in breast cancer has revealed Tfdp1p as one of the possible candidate target genes; in addition, tumors with high gene expression have been associated with markers of tumor proliferation and cell cycle progression (Melchor et al., 2009). Under normal physiological conditions and in most cancer cells, Tfdp1p is a predominant protein that binds to E2F; deregulated TFDP1/E2F1 is known to induce stress leading to high levels of p53 in cancerous cells (Zhan et al., 2017).

Foxm1p associated with Cluster 3 is an oncogenic transcription factor that is overexpressed in most human cancers. Foxm1p is involved in cell migration, invasion, angiogenesis, and metastasis. The important role of Foxm1p in cancer confirms its importance for therapeutic intervention (Halasi and Gartel, 2013). Understanding the regulation and function of Foxm1p has gained attention, which will provide potential roles of it in cancer and additional diseases.

Another TF associated with Cluster 3 is Cenpa (centromere protein-A), which was shown to be important in hepatocellular carcinoma development (Zhang et al., 2020), and gastric cancer progression and prognosis (Xu et al., 2020).

## 4. Conclusion

The analyses reported in this study have shown that doxorubicin causes common changes in gene expression in different types of cancer. Significant differential expressions were mainly observed in genes that have a role in transcription, regulation of transcription, and regulation of cell proliferation. Binding of protein, chromatin, and DNA are among significant molecular functions in addition to several terms related to RNA polymerase II activity, indicating that doxorubicin targets transcription machinery of RNA polymerase II, related to DNA intercalating property of doxorubicin. Further analysis of the PPI network revealed the interconnectivity of expression-level effects of doxorubicin on additional cancer types such as glioma, melanoma, thyroid, bladder, endometrial, and prostate cancers.

Identification of TFs within the significant clusters of the active network revealed the necessity of a comprehensive understanding of the regulation principles of these TFs in order to provide new potential therapeutic strategies for cancer therapy. As a result of this study, Pou5f1b, Znf428, Prmt3, Znf12, Erg, Tfdp1, Foxm1, and Cenpa can be suggested as new drug targets in the development of drugs that can be effective in application to different cancer types.

The common effects of doxorubicin on human cancer cells were investigated at the transcriptome and interactome levels and evaluated with an integrated systems biology approach. In more detailed studies in the future, the information on intracellular metabolite levels, metabolic flux values and protein amounts may be integrated and thus the mechanisms affected will be detailed. In addition, the results obtained in this study showed the applicability of the proposed systems biology approach to other drugs/drug types to obtain valuable information about the mechanism(s) of the drug in question. And in this way, new drug targets can be suggested as here.

## Supplementary Files for Doxorubicin-induced transcriptome meets interactome: identification of new drug targets

**Table S1:** Doxorubicin-induced transcriptome data used in the study: cancer type, cell line, GEO accession numbers of the control and case gene expression data

Cancer type	Cell Line	GEO control	GEO case
Renal cancer	786-0	GSM3233605 GSM3233606 GSM3233607	GSM3233608 GSM3233609 GSM3233610
Breast cancer	MCF7	GSM3233810 GSM3233811 GSM3233812	GSM3233815 * GSM3233816 * GSM3233817 *
Leukemia	CCRF-CEM	GSM3233659 GSM3233660 GSM3233661	GSM3233662 GSM3233663 GSM3233664
Non-small cell lung cancer	A549/ATCC	GSM3233623 GSM3233624 GSM3233625	GSM3233626 GSM3233627 GSM3233628
Colon cancer	COLO 205	GSM3233668 GSM3233669 GSM3233670	GSM3233671 GSM3233672 GSM3233673

* 100 nm of doxorubicin was administered.

**Table S2:** Differentially expressed common genes under doxorubicin treatment in different cancer types

ABCA2	CENPA	FOSL2	IGHG1	MED13L	PEX14	RPL13	TIAM1
ABHD5	COL6A1	FOXJ3	IRF1	MEF2C	PGM5	RUNX3	TLE6
ADAMTS7	CREBZF	FRYL	ITPR1	MGA	PHF14	SCAF4	TNFRSF10D
AGO2	CSF2	FYN	JARID2	MLLT10	PHLDA1	SCAPER	TNFSF9
AKAP13	CSHL1	FZR1	JMJD1C	MLLT3	PIM2	SETD2	TNIK
ALDOB	CTAG2	GABARAPL1	JUN	MSX2	PLCH1	SEZ6L	TNS3
ANKRD11	CXCL1	GADD45B	JUNB	MTHFD2L	PLEKHA5	SFI1	TRAF2
ANKRD12	CXCL3	GAS7	KCNAB2	MUM1	PMAIP1	SHB	TRIM36
APOE	CYLD	GATA2	KLF12	MYO10	PPM1H	SIPA1L1	TRIO
ARHGAP26	DAPK1	GDF15	LARGE	MYOZ2	PPP1R12B	SKAP2	TRPM3
ARL4C	DCT	GEMIN2	LCT	NAIP	PPP1R15A	SLC19A2	TSPAN5
ARMC9	DGCR14	GLP1R	LIF	NFATC4	PSD3	SLC5A3	UBR5
ATF5	DNAJB1	GPER1	LILRA5	NFIB	PSRC1	SOCS6	VAMP1
ATXN1	DNAJB9	GPR39	LIMCH1	NFKBIA	PTPRC	SOX2	VEZF1
BCAR3	DST	GPSM2	LINS1	NPAS2	PTPRO	SPATA2	WDR7
BCL11A	DUSP1	GRK5	LOC100287590	NPAT	RAB31	STK17B	XRCC4
BHLHE40	DUSP10	H2AFX	RUNX1	NR2E1	RALGPS1	SUPT7L	ZBTB14
BICD1	E2F1	HEXIM1	WNK1	NRF1	RAPGEF2	SVIL	ZBTB20
BNC2	ERBB4	HEY1	LOC643733	OGFOD2	RELB	SYNJ2	ZCCHC11
BTG2	ETV1	HIST1H1D	LPCAT4	PAQR6	RFX7	TAF1B	ZEB1
C16orf71	FAM120C	HIST1H1E	LRIG1	PARVA	RGS14	TBL1X	ZFYVE9
CAMK1	FAM168A	HLA-E	LTB4R	PATZ1	RGS2	TCF4	ZKSCAN1
CCNF	FAM193A	HOXD1	MAP3K14	PAX8	RHOH	TCF7L2	ZMYM1
CD59	FGF18	IER5	MAP3K5	PDE4DIP	RNF19B	TFAP2A	ZNF480
CEBPA	FN1	IFI44	MCAM	PELI1	RNF40	TFAP2B	

Figure S1 Active network constructed by protein-protein interactions of proteins encoded by the genes that are commonly differentially expressed under doxorubicin treatment in different cancer types

**Table S3:** Parameters of the active PPI (protein-protein interaction) network

Clustering coefficient :	0.045	Number of nodes :	7022
Connected components :	3	Network density :	0.001
Network diameter :	9	Network heterogeneity :	5.726
Network radius :	1	ISolated nodes :	0
Network centralization :	0.122	Number of self-loops :	0
Shortest paths :	49217254 (99%)	Multi-edge node pairs :	1309
Characteristic path length :	3.845		
Avg. number of neighbours :	4.120		

Figure S2 Clusters within the active PPI (protein-protein interaction) network. Clusters were identified via MCODE application in Cytoscape.

**Table S4:** Parameters of the clusters within the active PPI (protein-protein interaction) network

Cluster	Score (Density*Number of edges)	Number of nodes	Number of edges
1	4.152	80	170
2	3.565	24	44
3	3.412	18	36
4	3.333	4	8
5	3	3	5
6	3	3	3
7	3	5	6
8	2.667	7	8
9	2.667	4	5

Figure S3 Transcription factor (TF)-TF co-regulatory network for Cluster 2 (Right Side). Network is dynamically generated using the top results of the selected library (Left Side). Edges between TFs are defined by ChEA3 libraries and are directed where ChIP-seq supports the interaction.

Figure S4 Transcription factor (TF)-TF co-regulatory network for Cluster 3 (Right Side). Network is dynamically generated using the top results of the selected library (Left Side). Edges between TFs are defined by ChEA3 libraries and are directed where ChIP-seq supports the interaction.
