# Supplementary figures and images for: Doxorubicin-induced transcriptome meets interactome: identification of new drug targets
Source: Turk J Biol. 2021 Dec 20;46(2):137–44. doi: 10.3906/biy-2107-45 (PMC10393105; doi:10.3906/biy-2107-45)

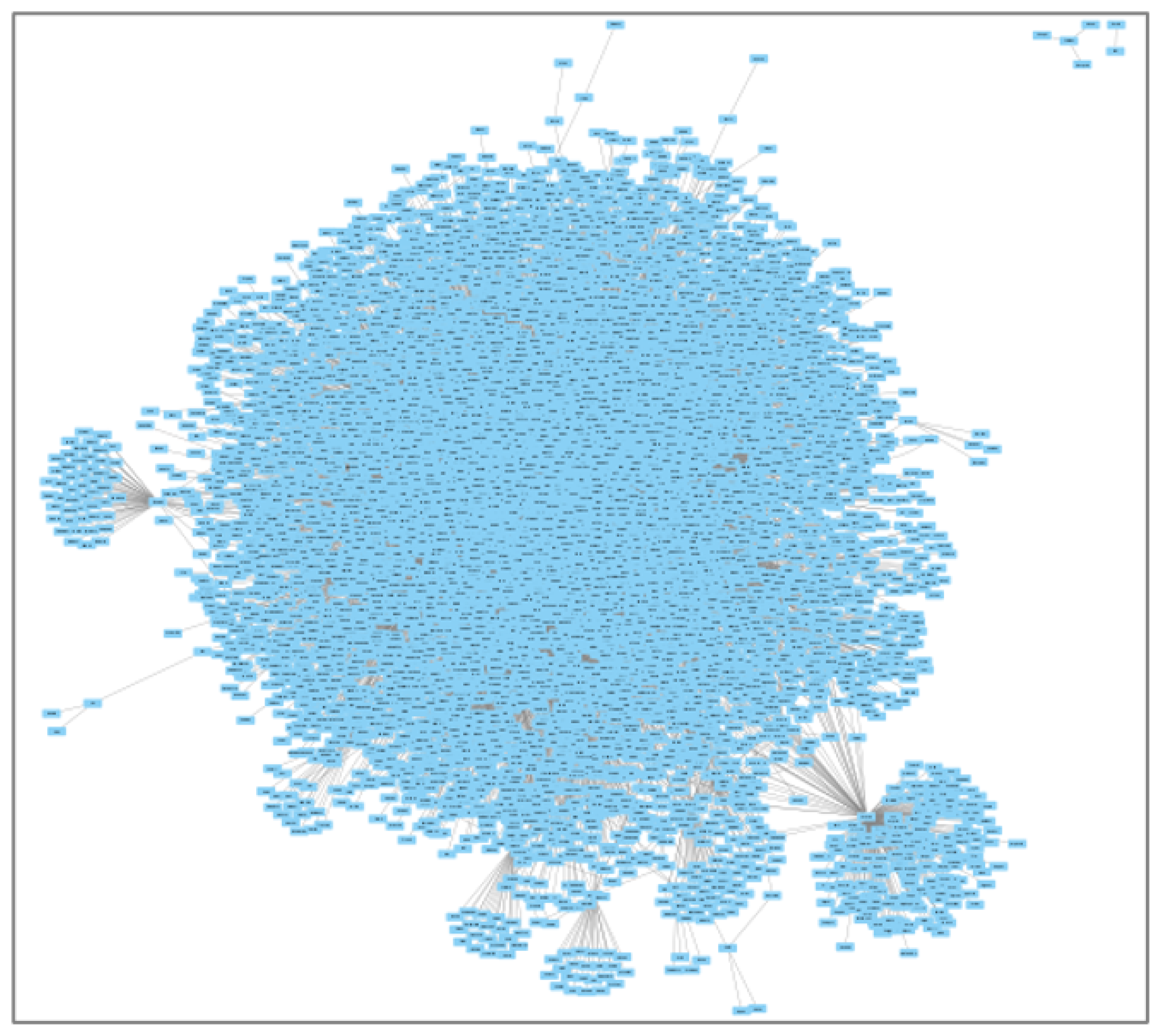

Supplement: Figure S1 — Active network constructed by protein-protein interactions of proteins encoded by the genes that are commonly differentially expressed under doxorubicin treatment in different cancer types [file turkjbiol-46-2-137s1.tif]

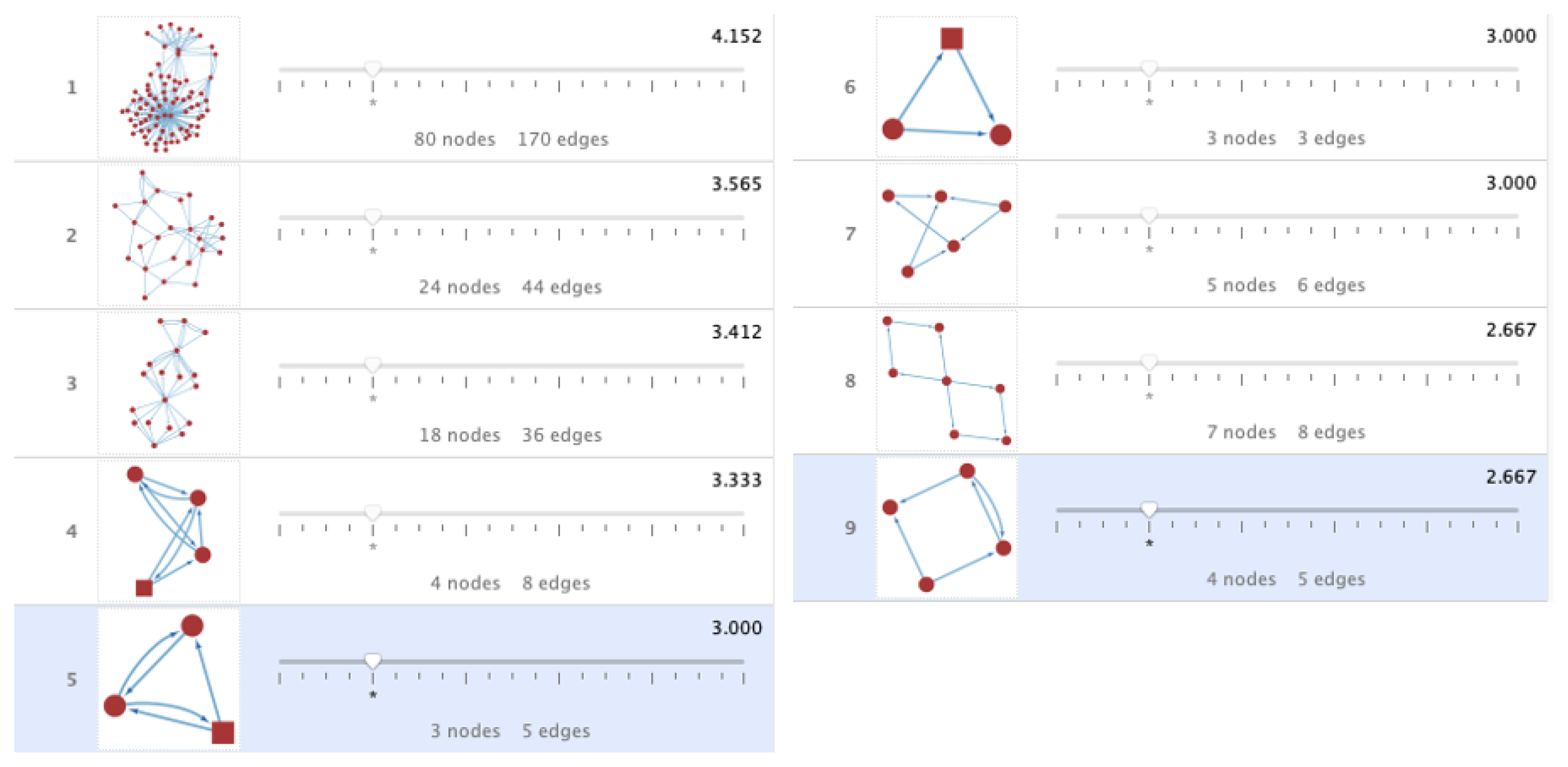

Supplement: Figure S2 — Clusters within the active PPI (protein-protein interaction) network. Clusters were identified via MCODE application in Cytoscape. [file turkjbiol-46-2-137s2.tif]

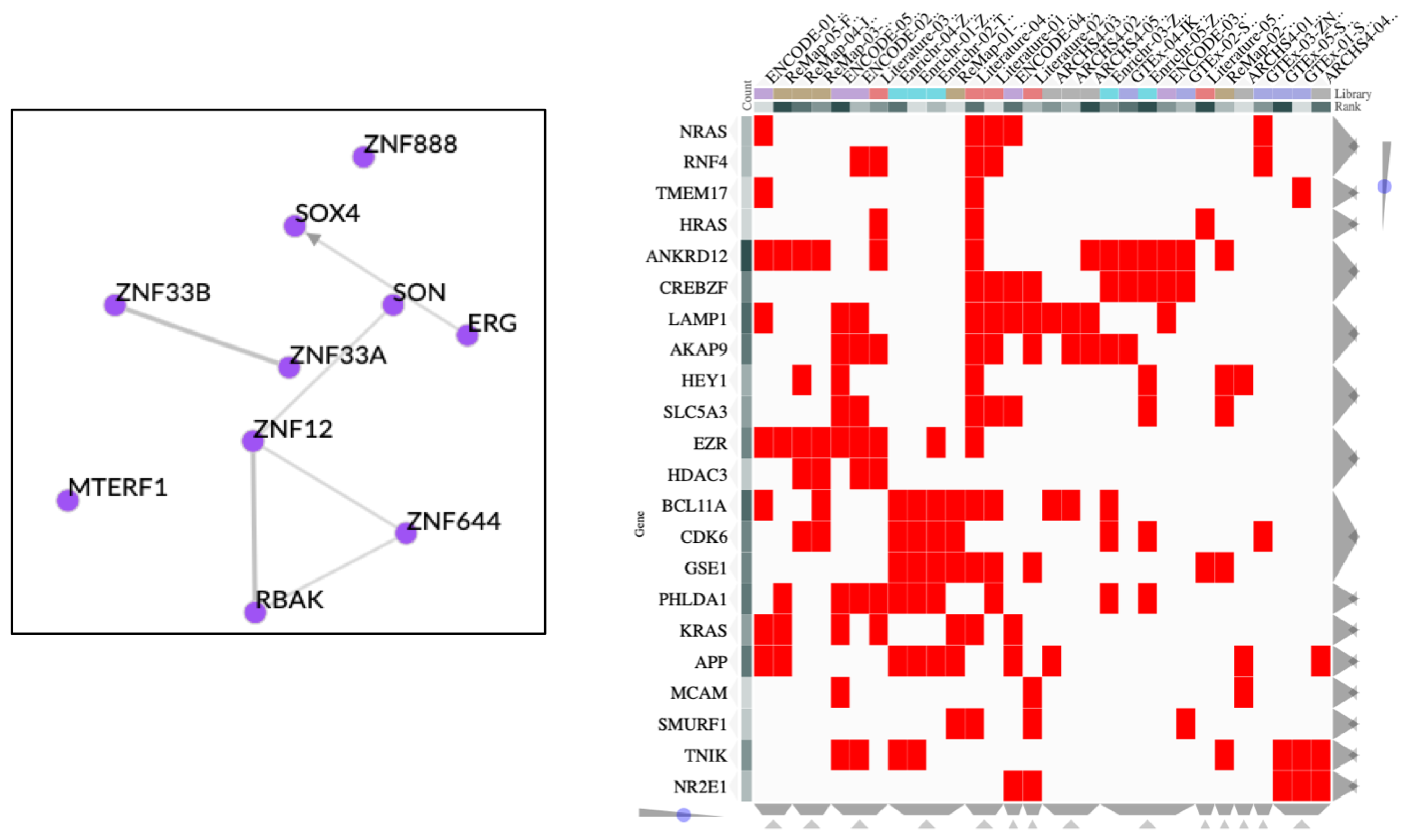

Supplement: Figure S3 — Transcription factor (TF)-TF co-regulatory network for Cluster 2 (Right Side). Network is dynamically generated using the top results of the selected library (Left Side). Edges between TFs are defined by ChEA3 libraries and are directed where ChIP-seq supports the interaction. [file turkjbiol-46-2-137s3.tif]

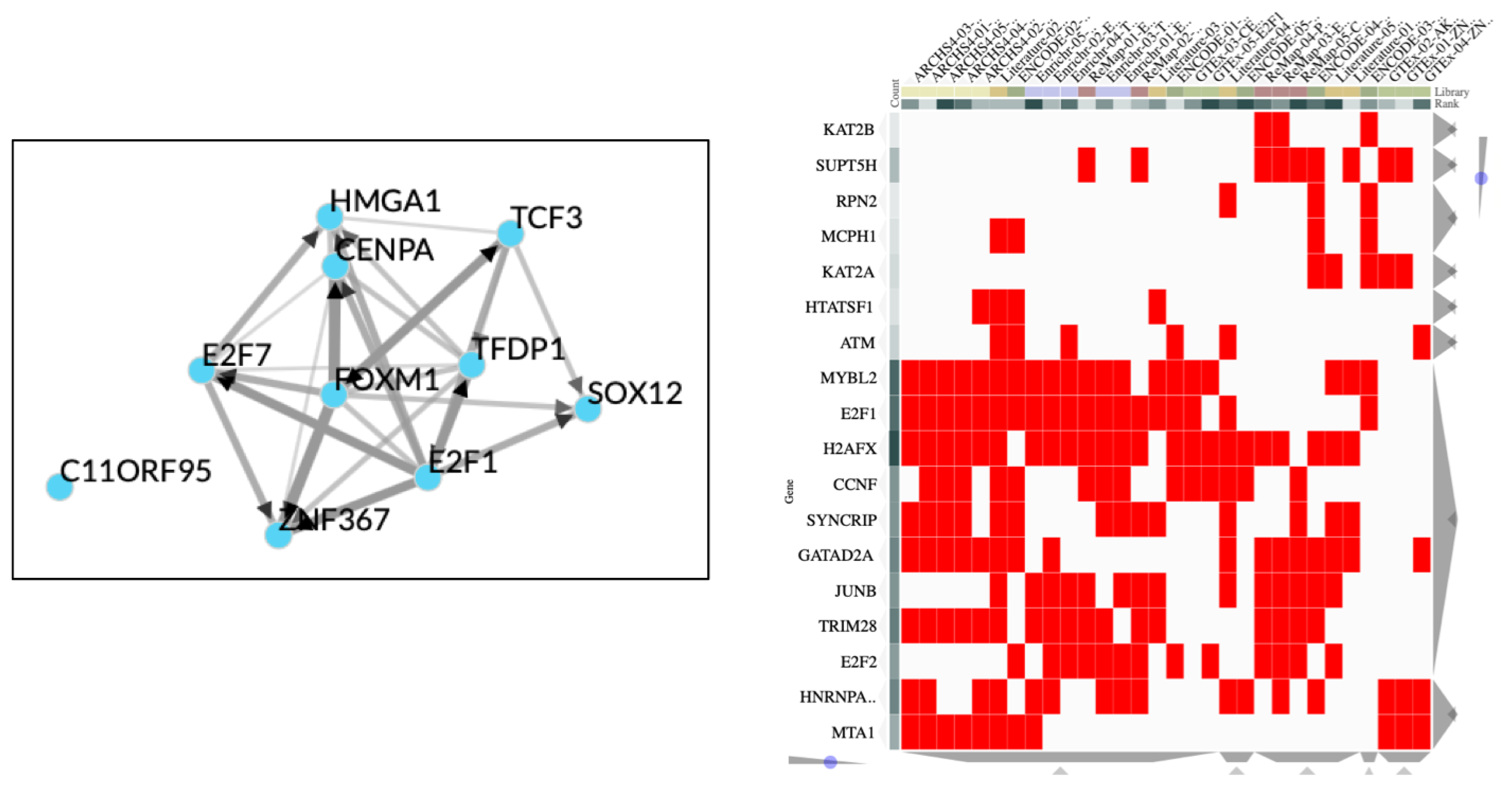

Supplement: Figure S4 — Transcription factor (TF)-TF co-regulatory network for Cluster 3 (Right Side). Network is dynamically generated using the top results of the selected library (Left Side). Edges between TFs are defined by ChEA3 libraries and are directed where ChIP-seq supports the interaction. [file turkjbiol-46-2-137s4.tif]
